# Adjuvants and Vaccines Used in Allergen-Specific Immunotherapy Induce Neutrophil Extracellular Traps

**DOI:** 10.3390/vaccines9040321

**Published:** 2021-04-01

**Authors:** Jasmine Karacs, Manuel Reithofer, Claudia Kitzmüller, Markus Kraller, Stefanie Schmalz, Sonja Bleichert, Johannes B. Huppa, Hannes Stockinger, Barbara Bohle, Beatrice Jahn-Schmid

**Affiliations:** 1 Department of Pathophysiology and Allergy Research, Medical University of Vienna, 1090 Vienna, Austria; jasmine.karacs@meduniwien.ac.at (J.K.); manuel.reithofer@meduniwien.ac.at (M.R.); claudia.kitzmueller@meduniwien.ac.at (C.K.); stefanie.schmalz@meduniwien.ac.at (S.S.); barbara.bohle@meduniwien.ac.at (B.B.); 2 Institute of Hygiene and Applied Immunology, Medical University of Vienna, 1090 Vienna, Austria; markus.kraller@meduniwien.ac.at (M.K.); johannes.huppa@meduniwien.ac.at (J.B.H.); hannes.stockinger@meduniwien.ac.at (H.S.); 3 Department of General Surgery, Medical University of Vienna, 1090 Vienna, Austria; sonja.bleichert@meduniwien.ac.at

**Keywords:** adjuvants, allergen-specific immunotherapy, aluminum hydroxide, MPLA, neutrophil extracellular traps, monocytes

## Abstract

Aluminum hydroxide (alum) and monophosphoryl-lipid A (MPLA) are conventional adjuvants in vaccines for allergen-specific immunotherapy (AIT). Alum triggers the release of neutrophil extracellular traps (NETs) by neutrophils. NETs contain expelled decondensed chromatin associated with granular material and may act as danger-associated molecular patterns and activate antigen-presenting cells. We investigated whether adjuvant-induced NETs contribute to innate responses to AIT-vaccines. Human neutrophils were incubated with alum, MPLA and adjuvant-containing AIT-vaccine preparations. NETs were verified by time-lapse and confocal fluorescence microscopy and quantitatively assessed by DNA and elastase release and ROS production. In contrast to MPLA, alum represented a potent trigger for NET release. Vaccine formulations containing alum resulted in less NET release than alum alone, whereas the vaccine containing MPLA induced stronger NET responses than MPLA alone. NETs and alum alone and synergistically increased the expression of molecules involved in antigen presentation, i.e., CD80, CD86 and CD83, by peripheral blood monocytes. Monocyte priming with NETs resulted in individually differing IL-1β- and IL-6-responses. Thus, NETs induced by adjuvants in AIT-vaccines can provide autonomous and cooperative effects on early innate responses. The high diversity of individual innate responses to adjuvants and AIT-vaccines may affect their therapeutic efficacy.

## 1. Introduction

Allergen-specific immunotherapy (AIT) elicits long-lasting clinical tolerance because it modifies the immune response to allergens [1,2]. As allergens *per se* are weak antigens, the vaccines used for subcutaneous AIT in Europe generally contain adjuvants, most commonly aluminum hydroxide (alum) [3]. Although used for more than 90 years, the operating mechanisms of this adjuvant are still not entirely clear. Its strong capacity to absorb proteins initially suggested a depot effect, however, the adjuvant effect wanes after a few hours [4]. As alum forms microparticles (1–10 μm) in aqueous environments, a particle effect promoting uptake and presentation of antigen by murine antigen-presenting cells (APC) has been demonstrated [5]. In addition, indirect effects via the release of danger-associated molecular patterns (DAMPs) have been discussed. These include urate crystals released from dying cells that activate the nucleotide oligomerization domain (NOD)-like receptor pyrin-domain-containing 3 (NALP3) inflammasome and caspase 1-dependent release of the mature forms of IL-1β and IL-18, which causes local inflammation [6,7,8,9,10,11]. Along these lines, alum was shown to activate the NALP3 inflammasome and IL-1β production in LPS-primed human PBMC [12]. Eventually, endogenous DNA released from host cells was found to be essential in the adjuvanticity of alum [13,14]. Lately, Stephen et al. found that extravasation and swarming of neutrophils as well as the release of neutrophil extracellular traps (NETs) early after injection of alum into mice was important for its adjuvanticity [15]. NETs are 3-dimensional web-like structures composed of decondensed chromatin and mitochondrial DNA that are complexed with toxic granular material such as myeloperoxidase (MPO), neutrophil elastase (NE) or the cationic cathelicidin peptide LL-37, which can keep microbes from growing and spreading [16,17]. We have recently demonstrated that injection of alum particles induces tissue infiltration by neutrophils and NET formation in a human ex vivo skin model [18]. 

After vaccine injection, neutrophils are the first cells recruited to sites of tissue damage, and subsequently promote the extravasation of monocytes [19]. These classical APC may be activated by NETs serving as DAMPs and initiate inflammation [20]. Alum has been shown to increase antigen-uptake and presentation in murine APC and to prolong their interaction with T cells in vitro [5]. Such T cell–APC interactions have also been observed in vivo and these were reduced by application of DNAse, suggesting an involvement of NETs [21]. In human monocytes, alum has been shown to promote differentiation by increasing the expression of HLA-class II and co-stimulatory molecules as well as markers of dendritic maturation [22,23,24]. Assuming that both NETs and alum are present at the injection site of AIT-vaccines, we sought to investigate their potential cooperative effects on the expression of surface molecules involved in antigen presentation or cytokine release. In addition, we included the second common adjuvant in AIT-vaccines, the toll-like receptor (TLR) 4 agonist 3-O-desacyl-4′-monophosphoryl-lipid A (MPLA), an analog of the lipid A moiety of lipopolysaccharide (LPS), which is applied together with microcrystalline L-tyrosine (MCT) [25,26,27]. In mice, MCT has shown similar immune activating potential as alum, but no Th2-bias [28]. LPS has been shown to induce NET formation in primed neutrophils [29], however, up to now MPLA has not been studied in this respect. In this study, we compared the capacity of the pure adjuvants and of AIT-vaccine preparations containing alum or MPLA to induce NETs in human neutrophils. Furthermore, we assessed the influence of NETs on allergen-uptake, expression of molecules involved in antigen presentation and cytokine production by monocytes.

## 2. Materials and Methods

### 2.1. Blood Donors

Peripheral blood was obtained from non-allergic and allergic individuals, who provided written informed consent before blood donation. None of the allergic donors had undergone AIT. This study conforms to the standards of the Declaration of Helsinki and had been approved by the Ethics Committee of the Medical University of Vienna (EK1488/2017). 

### 2.2. Neutrophil Isolation

Neutrophils were isolated from heparinized blood by Ficoll gradients, erythrocyte sedimentation and lysis of erythrocytes as described [18]. Obtained cell populations were >99% according to trypan blue staining and consisted of 92.4% ± 5.1 (mean ± SD; *n* = 14) CD16^+^CD66b^+^ neutrophils as evaluated flow cytometry. 

### 2.3. Stimulants

For stimulation experiments we used alum (Alu-Gel-S containing 2% aluminum hydroxide; SERVA, Heidelberg, Germany), ionomycin, phorbol-12-myristate-13-acetate (PMA), LPS (*E. coli* 1028:B12 (all from Sigma-Aldrich, St. Louis, MO, USA), ultrapure LPS, MPLA (both InvivoGen, San Diego, CA, USA), and the following allergoid AIT-vaccines: two alum-adjuvanted, Acaroid (A-HDM; containing house dust mite extract) and Allergovit (A-BP; containing birch pollen extract; both Allergopharma, Reinbek, Germany) and the MPLA/MTC-adjuvanted Pollinex Quattro (MT-GP; containing grass pollen extract, Bencard, Munich, Germany). For comparisons equivalent alum respectively MPLA concentrations were used.

### 2.4. Determination of Alum Concentrations with Lumogallion

To determine alum concentrations in vaccines, samples were incubated at RT for 16 h in the dark with 50 μM lumogallion (Santa Cruz Biotechnology, Santa Cruz, CA, USA) in aqua bidets. Alu-Gel-S containing 2% alum (SERVA, Heidelberg, Germany) was stained in parallel to create a standard curve. The stained alum particles and vaccines were centrifuged for 10 min at 13,000× *g*. The pellets were resuspended in HBSS medium and fluorescence measured using a TECAN Spark fluorescence reader (Tecan, Zürich, Switzerland). 

### 2.5. Time-Lapse Imaging 

Neutrophils (5 × 10^5^) were primed for 30 min in 300 μL HBSS containing 5 μM of the DNA-dye Sytox green (Thermo Fisher Scientific, Waltham, MA, USA) with 25 ng/mL GM-CSF in poly-D-lysine coated Lab-Tek^®^ chamber slides (Nunc, Thermo Fisher Scientific) at 37 °C and 5% CO_2_. Then, cells were incubated for 2 h either with ionomycin (5 μM), the indicated stimuli or medium alone as negative control. Imaging was performed every 60 s for up to 2 h with an inverted microscope (Eclipse Ti-E, Nikon, Tokyo, Japan), equipped with a 20x and 100x objective (CFI S Fluor 20x NA:0.75, Nikon). Data analysis was performed with ImageJ software. 

### 2.6. Preparation of Isolated NETs 

First, 6 × 10^6^ neutrophils were seeded into 6 mL HBSS in 6-well plates and NET formation was induced by incubation of the cells with 5 μM ionomycin for 4 h at 37 °C and 5% CO_2_. Since alum creates immense technological problems in isolating pure NETs, and we have demonstrated similar mechanisms in NET induction by alum and ionomycin [18], the latter was used as the NET-inducer for both procedures. NET formation was controlled by light microscopy. After 4 h of stimulation, the medium was carefully removed by pipetting, while the NET layer remained at the bottoms of the wells. NET layers were washed 3 times with PBS by careful pipetting without disruption of the NET layer. Then, NETs were partially digested by addition of 1 mL of 8 U/mL AluI (Thermo Fisher) in HBSS for 1 h at 37 °C and 5% CO_2_. After digestion by the restriction enzyme, NETs were lifted from the bottom by pipetting, collected and pooled in 15 mL tubes and centrifuged at 400× *g* for 5 min at 4 °C. The NET-DNA-rich supernatant was collected and the pellets containing some remaining neutrophils and mostly cell fragments were discarded. Long-size NETs isolated with AluI are thought to be close to physiological in vivo NETs [30]. The DNA concentrations in supernatants were determined by comparison with a DNA standard using Sytox orange. NETs were flash-frozen in liquid N_2_ and stored at −80 °C until used. 

### 2.7. Allergen-Uptake Experiments 

For allergen uptake experiments, recombinant Bet v 1 was used as model allergen and labeled with pHrodo, a pH-dependent dye that fluoresces in the acidic environment of endo-lysosomes, as described in [31]. Pilot experiments with different concentrations of Bet v 1 (1–6 μg/mL) identified 1 μg/mL as a suboptimal allergen concentration to be used in uptake experiments. Kinetic pilot experiments suggested evaluation of uptake after 1.5 h and 24 h, respectively. Then, 1 × 10^6^ PBMCs were pre-incubated in micronic tubes (MICRONIC, Lelystad, The Netherlands) for 1.5 h in 1 mL Ultra Culture medium with 100 ng/mL of isolated NETs (the optimum non-toxic concentration of NETs as determined in pilot experiments) and NET buffer or medium as controls at 37 °C and 5% CO_2_. Finally, 1 μg/mL Bet v 1-pHrodo, 30 min pre-adsorbed to 10 μg/mL of alum or Bet v 1-pHrodo alone was added and stimulated for 1.5 or 24 h. The medium served as negative control. 

### 2.8. Stimulation of PBMC with NET Layers and/or Alum or LPS

To create NETs at the bottom of wells for co-incubation with PBMC, 3 × 10^5^ neutrophils were seeded into 48-well plates and stimulated for 2 h with 5 μM ionomycin. The supernatant was removed and NET-layers washed 3 times with PBS. Next, 1 × 10^6^ PBMC in 1 mL AIM-V medium (Gibco, Thermo Fischer Scientific) were added to these NET layer and after 30 min of incubation at 37 °C and 5% CO_2_, PBMC were stimulated with alum at indicated concentrations, 1 μg/mL LPS (Sigma) or left with NETs for 24 h at 37 °C and 5% CO_2_. PBMC in AIM-V served as negative control. 

### 2.9. Confocal Fluorescence Microscopy 

A total of 1.5 × 10^6^ neutrophils were seeded on poly-D-lysine (Merck, Sigma-Aldrich, St. Louis, MO, USA) coated glass coverslips (13 mm) and primed with 25 ng/mL GM-CSF (PeproTech, Rocky Hill, NJ, USA) in 1 mL HBSS for 30 min at 37 °C and 5% CO_2_. Then, cells were stimulated for 3 h HBSS containing 5 μM ionomycin or with 5 μg/mL MPLA, 100 μg/mL alum, or 100 μg/mL of the following vaccines: A-HDM, A-BP and 2.25 μg/mL MT-GP. HBSS medium alone served as negative control. Cells were fixated in 4% paraformaldehyde in PBS and stained. All following steps were performed in a humid chamber at RT in wells made from parafilm containing about 100 μL of fluid. Coverslips were washed 3 times for 5 min in PBS. Cells were permeabilized in PBS containing 5 mM NH_4_Cl and 0.2% saponin (Sigma-Aldrich). Unspecific binding was blocked with 20% AB-serum in PBS for 60 min. The coverslips were incubated for 1 h with 1:400 polyclonal rabbit anti-myeloperoxidase (MPO) (Cell Marque Cooperation, CA, USA) or 1:500 monoclonal mouse anti-LL-37 (Santa Cruz Biotechnology, Dallas, Texas) antibodies in blocking buffer. After 3 washing steps for 5 min, coverslips were incubated for 1 h with 1:500 anti-rabbit Alexa Fluor 488 (Jackson Immuno Research Inc., West Grove, PA, USA) and anti-mouse Alexa Fluor 568 (Thermo Fisher Scientific). After washing, DNA was stained for 15 min with 5 μM of DRAQ5 (Invitrogen, Thermo Fisher Scientific). Imaging was performed with an Axiovert 200 microscope (Zeiss, Oberkochen, Germany) and Z-stacks analyzed by Volocity Software (Perkin-Elmer, Waltham, MA, USA). 

### 2.10. Quantification of Extracellular DNA 

Neutrophils (2 × 10^5^) in HBSS medium containing 25 ng/mL GM-CSF and the cell-impermeable DNA-dye Sytox orange (Thermo Fisher Scientific) in black flat bottom 96-well plates (Thermo Fisher) were incubated for 30 min, and then stimulated in triplicates with ionomycin (5 μM), alum, MPLA or the original vaccines at the indicated concentrations. Fluorescence was measured every 2 min for up to 3 h at 575 nm with a TECAN Spark reader. Areas under the curves (AUC) were calculated and compared to medium control resulting in fold changes. 

### 2.11. Detection of Mitochondrial and Cytoplasmic Reactive Oxygen Species (ROS) 

To assess mROS, 2 × 10^5^ neutrophils in black flat bottom 96-well plates in HBSS containing 25 ng/mL GM-CSF were pre-incubated for 1 h with 5 μM MitoSOX Red (Life Technologies, Carlsbad, CA, USA) at 37 °C. Then, cells were stimulated with ionomycin, alum, MPLA and different vaccines at indicated concentrations. mROS-induced fluorescence was measured as described [18]. To detect the production of cytosolic ROS (cROS), chloromethyl-2’7’-dichlorodihydrofluorescein diacetate (CM-H_2_CFDA) (Molecular probes, Life Technologies, Carlsbad, CA) was used; 1 × 10^6^ neutrophils were incubated for 30 min in 1 mL of 1 μM CM-H_2_CFDA in HBSS supplemented with 25 ng/mL GM-CSF at 37 °C and 5% CO_2_ in Falcon tubes. After washing with PBS and 30 min incubation with the indicated stimuli in HBSS in 24-well plates, the cells were harvested and fluorescence was measured immediately by flow cytometry on a BD FACS Canto II (BD Biosciences, Franklin Lakes, NJ, USA) and analyzed with FlowJo software (BD Biosciences).

### 2.12. Quantification of Neutrophil Elastase 

First, 5 × 10^6^ neutrophils were primed with 25 ng/mL GM-CSF in DMEM without phenol red in 24-well plates and then incubated for 3 h with 5 μM ionomycin, 25 nM PMA, alum, MPLA or the original vaccines at the indicated concentrations. Then, 500 mU/mL MNase (Sigma-Aldrich) was added and incubated for 30 min at 37 °C. The supernatants were harvested and remaining cells removed by centrifugation at 400× *g* for 5 min. The supernatants were collected and elastase activity was quantified colorimetrically. Conversion of 100 μM substrate N-(methoxysuccinyl)-Ala-Ala-Pro-Val 4-nitroanilide (Sigma-Aldrich) into a yellow product within 15 min at RT was measured using a TECAN Spark plate reader. Fold-changes were calculated by comparison to medium controls. 

### 2.13. Flow Cytometry 

Non-specific binding sites on harvested cells were blocked with 20% inactivated AB-serum in PBS containing 0.1% BSA and 0.1% NaN_3_ for 20 min at 4 °C. Then, cells were stained for 30 min at 4 °C with fluorescence-labelled antibodies: CD83-FITC, CD19-PECy7, CD80-APC647, CD14-APCCy7, HLA-DR-Brilliant Violet 421 (all Biolegend, San Diego, CA) CD86-PE, CD86-PECy7 (both BD Biosciences) and fixable viability dye eFluor506 (eBioscience, San Diego, CA, USA). Fluorescence was assessed by flow cytometry on a BD FACS Canto II and analyzed with FlowJo software.

### 2.14. Analysis of Cytokines

To analyse cytokines releases in SN, PBMC were plated in 48-well plates at a density of 2 × 10^6^ cells per well in 500 μL DMEM containing 10% FCS. Cells were either primed with 25 pg/mL of ultrapure LPS (InvivoGen), 100 ng/mL of isolated NETs, or were left untreated for 3 h. Then cells were stimulated for 6 h with either increasing doses of alum or the complete vaccines at indicated concentrations. Supernatants were collected and assayed for IL-1β and IL-6 with ELISA kits (R&D Systems, Minneapolis, MN, USA) according to the manufacturer’s instructions. Detection limits were 3.91 pg/mL for IL-1 β and 9.4 pg/mL for IL-6. 

### 2.15. Statistics

Data were analyzed using GraphPad Prism 5 (GraphPad Software, Inc., La Jolla, CA, USA) Statistical significances were determined by using one-way ANOVA for repeated measurements followed by Dunnett’s post tests. Differences were considered as statistically significant for values of *p* ≤ 0.05.

## 3. Results

### 3.1. Adjuvants and Vaccines Induce NETs in Isolated Human Neutrophils

**Figure 1:**
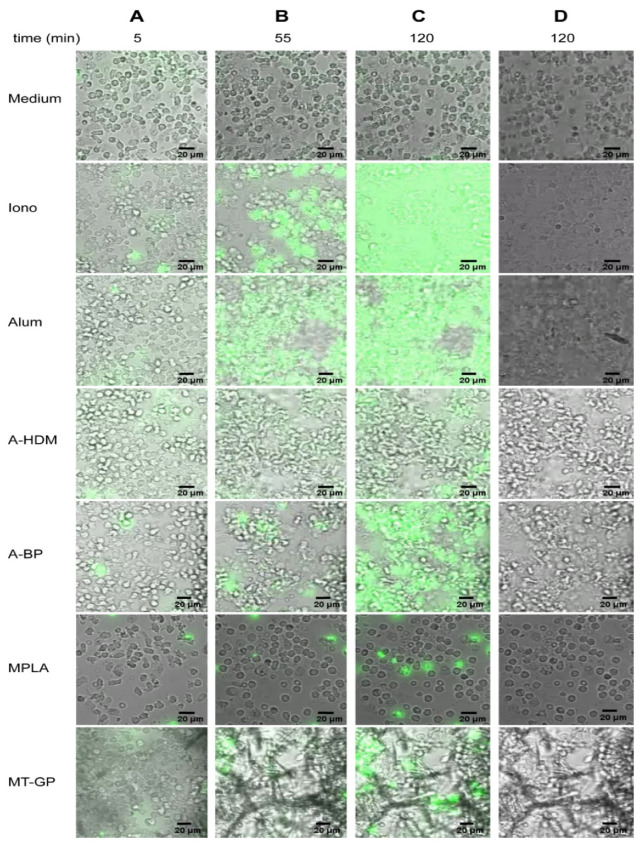
Time lapse imaging of neutrophil extracellular trap (NET) release. Freshly isolated human neutrophils were primed with 25 ng/mL of GM-CSF and incubated for 2 h with medium, ionomycin (Iono; 5 μM), alum (100 μg/mL), the alum containing vaccines A-HDM (89 μg alum/mL) and A-BP (100 μg alum/mL), and further monophosphoryl-lipid A (MPLA; (5 μg/mL) and the vaccine containing MPLA and L-tyrosine microcrystals (represented by the needle-like structures): MT-GP (2 μg MPLA/mL) in the presence of Sytox green. Shown images were acquired via wide field microscopy at indicated time points (**A**–**D**) with merged channels showing DNA staining (green) (**A**–**C**) compared to bright field alone (**D**). HDM, house dust mite extract; BP, birch pollen extract; GP, grass pollen extract. (Scale bars, 20 μm).

Human neutrophils were incubated for 2 h in the presence of the cell impermeable DNA dye Sytox green and the characteristic process of NET formation was monitored by live cell imaging. An overview featuring different time points from recorded videos is shown in Figure 1. When placed in medium alone the cells stayed intact, moved intensely and did not adhere (Figure 1, Videos S1 and S4). Stimulation with the positive control ionomycin induced typical features of NET formation [20,32], such as adherence and flattening, followed by chromatin decondensation, cell swelling, membrane rupture and DNA extrusion into the extracellular space (Figure 1; Videos S2 and S5). Alum-stimulated neutrophils showed similar features, however, the released DNA did not diffuse, but formed defined structures (Figure 1; Videos S3 and S6), most probably by binding to alum aggregates. The alum-containing vaccines A-HDM and A-BP induced similar morphological changes, but activated lower numbers of neutrophils than pure alum (Figure 1; Videos S7 and S8). MPLA, at a concentration that was comparable to the vaccine, did not induce NET formation. Intracellular DNA staining with Sytox green indicated increased membrane permeability (Figure 1, Video S9), but only very high MPLA concentrations (100 μg/mL) led to visible release of DNA (data not shown). Stimulation with the MPLA-adjuvanted vaccine MT-GP led to cell accumulation as with alum or ionomycin and Sytox green positive areas (Figure 1, Video S10). However, a more precise morphological evaluation was impeded by the needle-like MCT particles, which are also contained in the vaccine preparations. 

**Figure 2:**
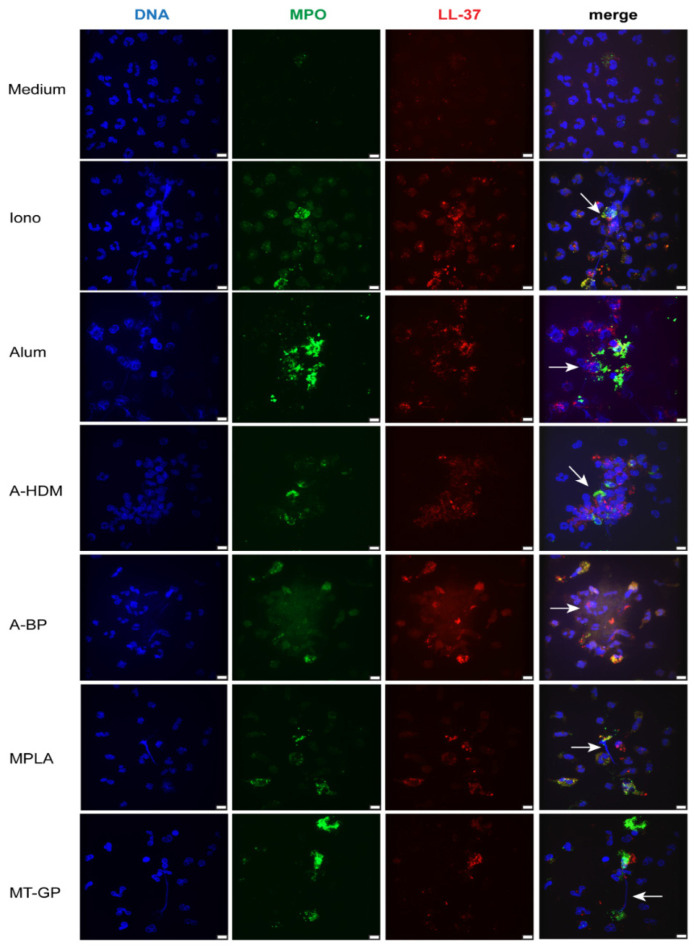
Induction of NETs by adjuvants and complete allergen-specific immunotherapy (AIT)-vaccines. Human neutrophils were primed with 25 ng/mL GM-CSF and incubated for 3 h with medium, ionomycin (Iono; 5 μM), alum (100 μg/mL), the alum-adjuvanted vaccines (100 μg/mL), MPLA (5 μg/mL) and MT-GP (2.25 μg/mL). Co-localization of released DNA (blue) with the granular components MPO (green) and LL-37 (red) indicating NET formation is shown by confocal fluorescence microscopy. (Scale bars, 8 μm).

Next, co-localization of granular components and extracellular DNA was evaluated by confocal fluorescence microscopy (Figure 2) to unequivocally identify NETs. After 3 h of incubation, medium controls showed intact neutrophils with typical lobular nuclei and intracellular staining of MPO and the cathelicidin peptide LL-37. As expected, ionomycin-stimulated neutrophils displayed disintegrated nuclei or extracellular DNA associated with granular MPO or LL-37. Alum triggered a combined release of DNA and granular material, which attached to larger structures, most likely agglomerates of alum particles. Alum-adjuvanted vaccines (A-HDM, A-BP) had a similar effect as alum alone. MPLA- and MT-GP-stimulated cultures contained mainly intact neutrophils and few extracellular DNA-fibers decorated with granular material.

### 3.2. Quantification of NET Release

**Figure 3:**
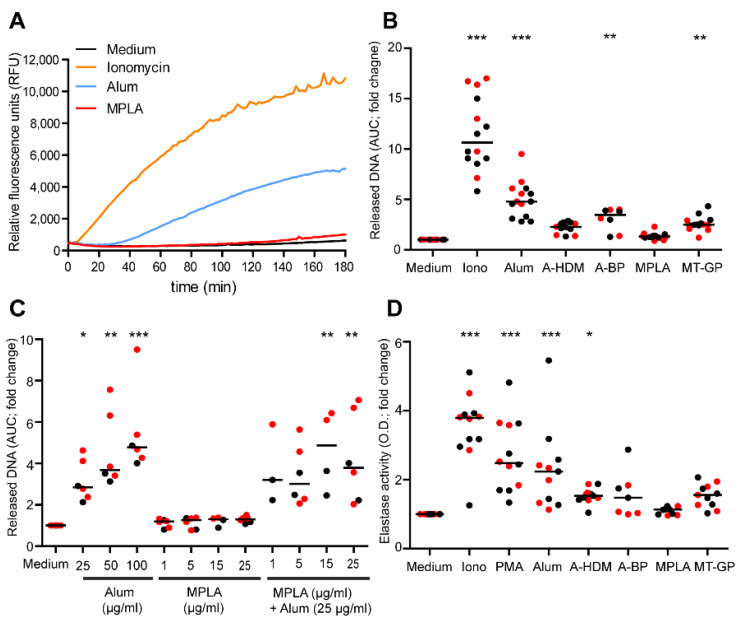
Quantification of NET release. Human neutrophils were primed with 25 ng/mL GM-CSF and incubated for 3 h with medium, ionomycin (Iono; 5 μM), alum (100 μg/mL), A-HDM (100 μg alum/mL), A-BP (100 μg alum/mL), MPLA (5 μg/mL) and MT-GP (2.25 μg MPLA/mL). The kinetics and quantification of DNA release was assessed by fluorescence of the cell impermeable DNA dye Sytox orange in plate reader assays. (**A**) One representative experiment and (**B**) cumulated data of 14 independent experiments using neutrophils from different donors. (**C**) Co-stimulation of neutrophils with MPLA and alum in 5 independent experiments with different donors. (**D**) Neutrophil elastase in the supernatant of MNase-treated cultures 3 h after stimulation with the indicated substances was quantified colormetrically with N-methoxysuccinly-Ala-Ala-Pro-Val-p-nitroanilide as substrate (*n* = 12). (**B**,**C**) Areas under the curves (AUC) or (**D**) optical densities (O.D.) were measured and fold changes calculated relative to medium controls. Red dots indicate allergic donors. Horizontal lines show medians. Statistical significance was calculated by one-way ANOVA for repeated measurements followed by Dunnett’s post tests (* *p* < 0.05; ** *p* < 0.01; *** *p* < 0.001).

We measured the kinetics of DNA release by using the cell-impermeable DNA dye Sytox orange in a fluorescence plate reader assay. Similar to ionomycin, but with 20 min delay, alum triggered concentration-dependent DNA release starting about 30 min after stimulation (Figure S1A, Figure 3A,B). As neutrophils isolated from allergic subjects have been shown to possess a reduced capacity for NOX2-dependent, PMA-induced NET formation when compared to non-allergic subjects [33], we investigated whether NET formation induced by alum also differed in these groups. Stimulation with either ionomycin or alum induced similar DNA releases, implying that allergic subjects are not deficient in NOX2-independent NET release (data not shown). Both alum-adjuvanted vaccines led to lower levels of DNA release compared to matching concentrations of pure alum, and A-BP induced slightly higher amounts of released DNA than A-HDM (Figure 3B). MPLA alone did not cause measurable DNA release (Figure 3A,B), not even when applied at doses up to 50 μg/mL (Figure S1B). Interestingly, the MPLA-adjuvanted vaccine MT-GP induced measurable levels of DNA release (Figure 3B), possibly due to the MCT particles present in the vaccine. In house-prepared extracts alone did not induce any DNA-release by neutrophils (Figure S2). The combination of varying concentrations of MPLA (1–25 μg/mL) with alum did not result in synergistic effects, neither with a suboptimal concentration of alum (25 μg/mL) (Figure 3C), nor with higher amounts of alum (data not shown).

To substantiate the DNA release data by a second parameter, extracellular neutrophil elastase (NE) activities were determined in supernatants of isolated NETs. Significant NE activity was observed after stimulation with PMA, ionomycin or alum (Figure 3D). The alum-adjuvanted vaccines induced measurable NE, but less than alum alone. Also corroborating the DNA release data, MPLA did not induce substantial NE activity, whereas the MPLA-adjuvanted vaccine MT-GP did. 

### 3.3. Involvement of Reactive Oxygen Species (ROS) in NET Release

**Figure 4:**
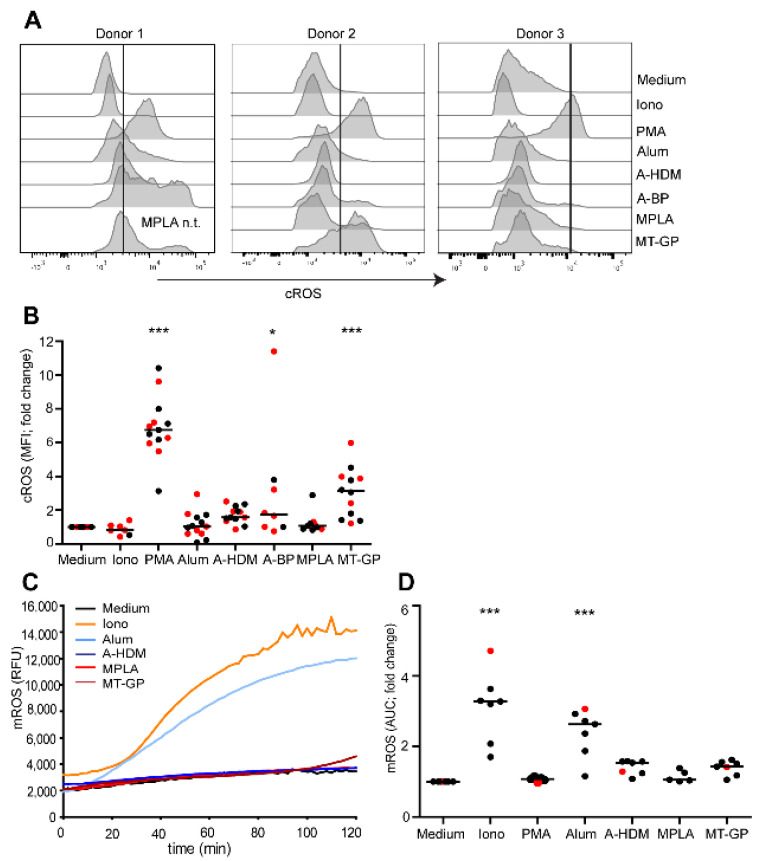
Involvement of ROS in NET release. Neutrophils were incubated with the indicated stimuli in the presence of the mROS indicator MitoSOX red and mROS-induced fluorescence was measured by a plate reader. (**A**) One representative experiment and (**B**) cumulative data of 9 independent experiments with different donors. Areas under the curve (AUC) and fold changes were calculated relative to medium controls. Horizontal lines show medians. (**C**,**D**) Neutrophils were loaded for 30 min with an indicator for cROS production (CM-H_2_DCFDA) and stimulated for 30 min with medium, ionomycin (Iono; 5 μM), PMA (25 nM), alum (100 μg/mL), A-HDM (100 μg alum/mL), A-BP (100 μg alum/mL), MPLA (5 μg/mL) and MT-GP (1 μg MPLA/mL). cROS production was assessed by flow cytometry. (**A**) 3 representative experiments from 3 different donors and (**B**) cumulative data of 9 independent experiments with different donors. Red dots indicate allergic donors. n.t., not tested Statistical significance was calculated by one-way ANOVA followed by Dunnett’s post tests (* *p* < 0.05; *** *p* < 0.001).

Two different pathways of NET induction exist, a NADPH-oxidase 2 (NOX2)- dependent pathway involving cytosolic (c)ROS as induced by PMA or lipopolysaccharide [34,35], and a NOX2-independent pathway involving mitochondrial (m)ROS as induced, e.g., by ionomycin or alum [18,35,36]. Production of NOX2-dependent cROS in neutrophils was measured by flow cytometry using the cell-permeant fluorescent probe CM-H_2_DCFDA, and NOX2-independent mROS were assessed with MitoSox Red using a plate reader. As expected, PMA stimulated the production of cROS (Figure 4A,B), and ionomycin the production of mROS exclusively (Figure 4C,D). Alum induced high levels of mROS, as did A-HDM but to a lower extent (Figure 4C,D). In contrast to MPLA, the vaccine MT-GP induced significant increases of cROS (Figure 4A,B). Unexpectedly, the alum-containing vaccines A-HDM and especially A-BP also induced cROS in some donors (Figure 4A,B), indicating that the allergen extract may have contained NOX2-activating substances. Similarly, MT-GP induced increased mROS in several subjects, which may be attributed to the MCT particles (Figure 4D). 

### 3.4. Influence of NETs and Alum on Allergen Uptake by Monocytes

**Figure 5:**
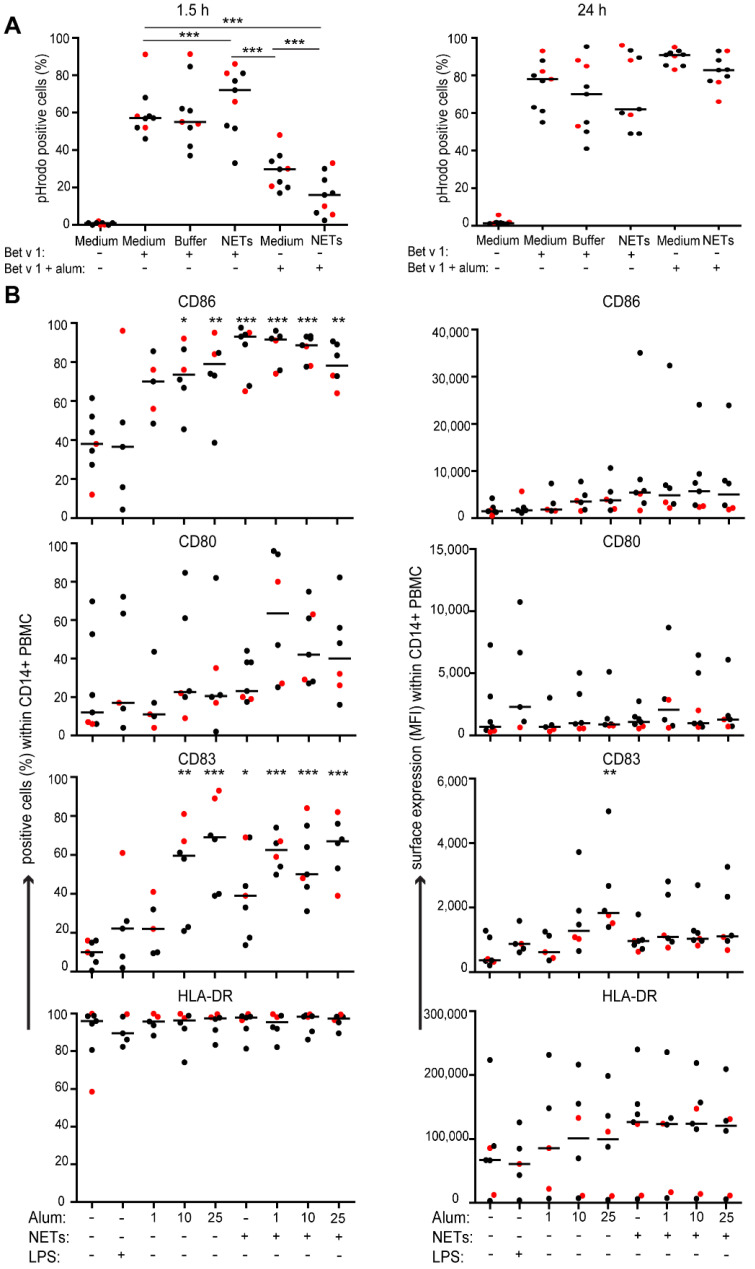
Effects of alum and NETs on antigen uptake and surface molecules involved in antigen-presentation. (**A**) Peripheral blood mononuclear cells (PBMC) were pre-incubated for 1.5 h with isolated NETs (100 ng/mL), NET buffer or medium alone. Then pHrodo-labelled Bet v 1 (1 μg/mL) or Bet v 1-pHrodo (1 μg/mL) that had been pre-adsorbed to alum (10 μg/mL) for 30 min was added. The percentages of CD14^+^Bet v 1-pHrodo^+^ cells were quantified after 1.5 h (left panel) and after 24 h (right panel) by flow cytometry. Cumulative data of 9 independent experiments with 9 different donors are shown. In Figure S4A one representative example is shown. (**B**) PBMC were incubated for 24 h with either medium, LPS (1 μg/mL), pre-formed NET layers, or increasing doses of alum (1, 10, 25 μg/mL) or alum in combination with NET layers, respectively. The percentages of positive cells within the CD14^+^ population and the mean fluorescence intensities (MFI) were quantified by flow cytometry for CD86, CD80, CD83 and HLA-DR. Cumulative data of 7 independent experiments with 7 different donors are shown. Red dots indicate allergic donors. Horizontal lines show medians. One-way ANOVA followed by Dunnett’s post tests (* *p* < 0.05; ** *p* < 0.01; *** *p* < 0.001).

As alum and alum-adjuvanted vaccines induced NETs, their effect in combination with alum on allergen-uptake by CD14^+^ monocytes was investigated using pHrodo-labelled Bet v 1, the major birch pollen allergen (Figure S3 and Figure 5A). After 1.5 h, the percentages of Bet v 1^+^CD14^+^ cells were similar in medium and NET isolation buffer control (Figure 5A). Pre-incubation with NETs resulted in a slight, but not significant increase. Uptake of Bet v 1 pre-adsorbed to alum was significantly lower compared to soluble Bet v 1. After 24 h, however, 91% of monocytes incubated with Bet v 1 pre-adsorbed to alum were Bet v 1^+^. Pre-incubation of cells with NETs tended to decrease the uptake of allergen at this time-point.

### 3.5. Effect of NETs and Alum on the Expression of Surface Molecules Involved in Antigen Presentation

Next, we examined whether NETs would modify expression of molecules involved in antigen presentation. PBMC pre-incubated with NETs were stimulated with different concentrations of alum for 24 h and the expression of co-stimulatory molecules CD80 and CD86 and CD83, a molecule indicating dendritic cell maturation of monocytes [37], on CD14^+^ monocytes was analyzed. Although large inter-individual differences were observed, expression of CD86 and CD83 were significantly enhanced (Figure 5B). Both, alum and NETs alone already increased the percentages of positive cells, and at a low alum concentration, NETs promoted an additional increase of CD80, CD86 and CD83. NETs or alum also augmented the mean fluorescence intensity (MFI) of HLA-DR without reaching significance, but their combination had no additional effect. 

### 3.6. Effect of NETs on IL-1β- and IL-6 Production

**Figure 6:**
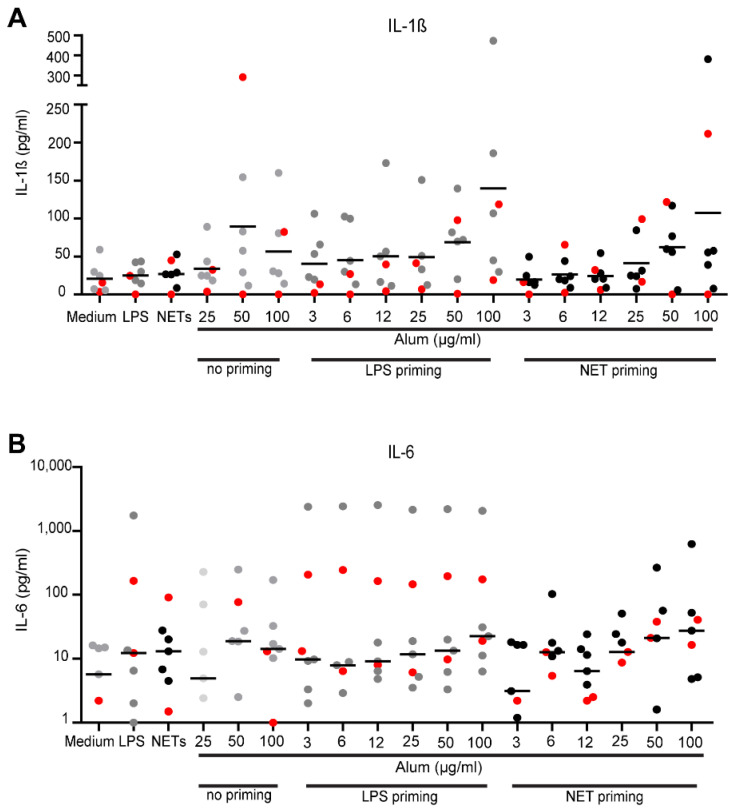
IL-1β and IL-6 production in PBMC induced by lipopolysaccharide (LPS)- or NET- priming and alum stimulation. PBMC were incubated for 3 h with either medium (neg. control), ultrapure LPS (25 pg/mL) or isolated NETs (100 ng/mL) and then stimulated for 6 h with increasing doses of alum (3, 6, 12, 25, 50 and 100 μg/mL). Cytokine levels of (**A**) IL-1β and (**B**) IL-6 in SN were measured by ELISA. Cumulative data of 3 independent experiments with 7 different donors are shown. Red dots indicate allergic donors. One-way ANOVA followed by Dunnetts post tests.

NETs induced by cholesterol crystals have been shown to prime murine and human monocytes to release IL-1β after subsequent stimulation with these crystals [38].Therefore, we tested whether NETs primed PBMC to release IL-1β upon subsequent stimulation with alum or vaccine preparations. Medium and ultrapure LPS served as negative and positive controls for priming. Striking inter-individual differences were obtained (Figure 6A). Unprimed PBMC produced IL-1β in response to higher doses of alum, similar to PBMC primed with ultra-pure LPS. Priming with NETs also induced an elevated production of IL-1β. Interestingly, when compared individually, priming with LPS or NETs led to 2 to 4.5-fold increased IL-1β production compared to alum alone in different donors. For IL-6, which represents another pro-inflammatory cytokine but is produced independently of NALP3 activation, similar, even less consistent data were gathered (Figure 6B). 

**Figure 7:**
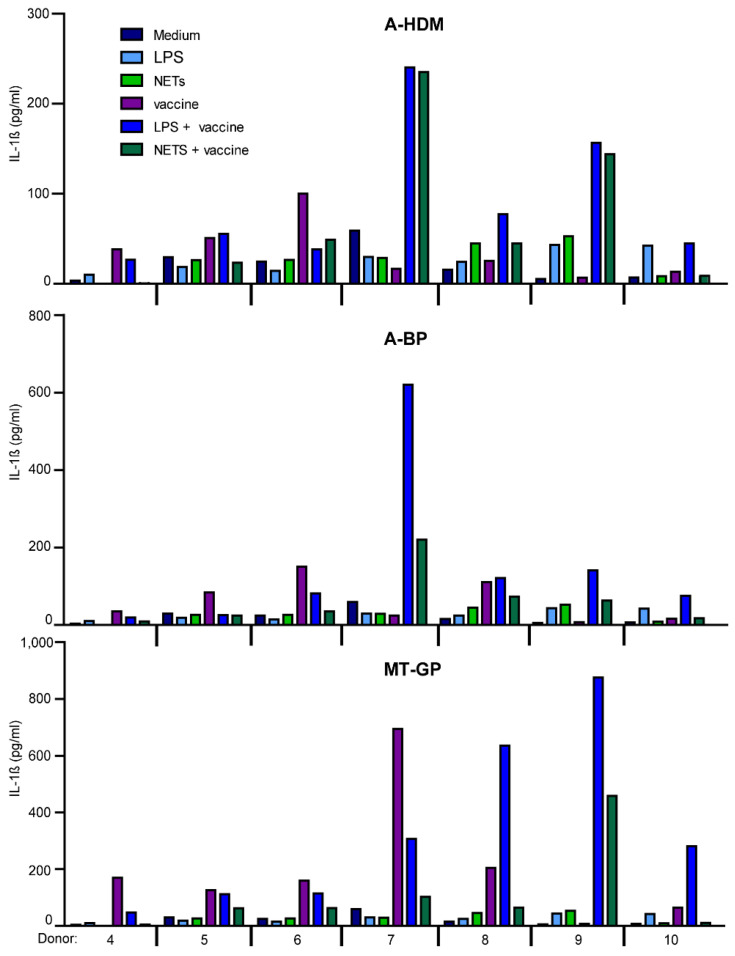
Induction of IL-1β in PBMC by LPS- or NET-priming and stimulation with complete vaccines. PBMC were incubated for 3 h with either medium (neg. control), ultrapure LPS (25 pg/mL) or isolated NETs (100 ng/mL) and then stimulated for 6 h with either one of the alum-adjuvanted vaccines (A-HDM, A-BP) or the MPLA-adjuvanted one MT-GP. Cytokine levels of IL-1β in SN were measured by ELISA. Cumulative data of 4 independent experiments with 7 different donors are shown. Subjects No. 4 and No. 7 were allergic.

Priming of PBMC with LPS or NETs prior to stimulation with AIT-vaccines also resulted in large inter-individual differences not leading to statistical significant differences (Figure S4 and Figure 7). MT-GB induced the highest levels of both cytokines. Pre-treatment with LPS in four donors led to increased IL-1β production, while NETs in most cases led to reduced levels. PBMC of some donors reacted with 3 to 118-fold IL-1β production after LPS-priming, while others were primed with NETs or LPS (8 to 62-fold) or were not primed by LPS or by NETs (Figure 7).

## 4. Discussion

It has been demonstrated that after alum-adjuvanted vaccination, neutrophils infiltrate the tissue and recruit monocytes to the injection site [19]. As alum induces NETs within a short time [18], we hypothesized that the simultaneous presence of alum particles and NETs may have a cooperative effect on the early infiltrating monocytes. In addition to pure adjuvants, we assessed AIT-vaccine preparations for their capacity to induce NETs and their influence on antigen-presenting cells in vitro.

First, we verified that alum and alum-containing vaccines induce NET release by monitoring morphological changes and DNA expulsion by neutrophils and by colocalization of granular components and extracellular DNA. Interestingly, vaccine preparations including allergen sources and alum were less efficient than alum alone. Possibly, the positive charge of alum, which is a major factor in its NET induction [18], was saturated by substances present in the allergen extracts. MPLA, on the other hand, induced only a sparse release of DNA fibers in neutrophils. In line with this, a recent review on LPS-induced NET formation revealed that LPS only poorly induces NETs [39]. Therefore, previously reported LPS-induced NET release may refer to contaminating ligands of additional receptors of innate immunity such as TLR2.

NET formation is either propagated by NOX2-dependent cROS or NOX2-independent mROS. Alum induces mROS and LPS cROS [18,35]. Interestingly, the alum-adjuvanted and MPLA-containing vaccines induced a mixed mROS and cROS response. Thus, alum as well as tyrosine particles in the vaccines triggered mROS production, supposedly via lysosomal destabilization in neutrophils [18]. We wished to test the particle effect of MCT, however, we failed to obtain the pure substance. On the other hand, allergen extracts in alum-adjuvanted vaccines contained components that activated cROS production in some individuals, suggesting stimulation of NOX2 via innate pattern recognition receptors on neutrophils. At concentrations present in AIT-vaccines, the TLR4 agonist MPLA did not induce appreciable amounts of cROS, explaining its minimal NET-inducing capacity. In order to optimize the efficacy of vaccination, MPLA in combination with alum has been developed in the adjuvant system AS04, which is currently administered as part of human papilloma and hepatitis virus vaccines [40]. However, with regard to NET-induction, no synergistic effect of MPLA and alum was observed.

In accordance with previous reports [22,23,24], alum promoted monocyte differentiation towards the dendritic cell pathway indicated by increased expression of the co-stimulatory molecules CD80 and CD86 and CD83 [37,41]. Co-engagement of CD83 ligand, assumed to bind homotypically in trans to CD83 expressed on recently activated T cells- with TCR and CD28, has not only been reported to support expansion and long-term survival of newly primed CD8^+^ T cells, but also to promote CD4^+^ T cell responses [41,42]. Increases of CD86 and CD83 have been found to be alum-specific, but neither are provoked by latex beads, other phagocytosed mineral adjuvants, emulsions or by LPS [24,43]. Notably, NETs also significantly increased the expression of CD80, CD86 and CD83, which was further promoted by low concentrations of alum. Both alum and NETs contain strongly positively charged molecules, which may cause similar effects. Notably, Barrientos et al. reported that NETs, which had been produced in a similar way as in this study, had no effect on human monocyte-derived dendritic cells *per se,* however, they significantly reduced LPS-induced maturation of these cells [30]. This observation together with our data suggests that a pro-inflammatory effect of NETs depends on the differentiation state of monocyte-derived APC.

While NETs increased the uptake of soluble allergen into monocytes within a short time, the apparent uptake of alum-adsorbed allergen peaked after 18–24 h (data not shown). The latter observation is in line with a previous in vivo study indicating that alum not only increases antigen uptake, but also retards antigen degradation resulting in sustained antigen-presentation in vitro and in vivo [5,21]. Interestingly, when applied together, NETs led to decreased alum-mediated allergen uptake. We consider it likely that the positively charged molecules in NETs compete with alum for protein binding. Binding to NETs and joint uptake would be in line with a NET-promoted increase in uptake of soluble allergen.

As human monocytic cells do not express TLR9, the mechanism of how they are stimulated by NETs remains to be identified. The positively charged LL-37, which is present in NETs, has been shown to be able to transfer extracellular self-DNA into monocytes, where it induced interferon-β in a TLR-independent manner involving the cGAS/STING/TBK1 signaling pathway [44]. Uptake of oxidized mitochondrial DNA by PBMCs without transfectant and induction of pro-inflammatory cytokines has been reported [45]. Along this line, it has been shown that in the presence of NFkB (after priming e.g., by LPS, GM-CSF), oxidized mitochondrial DNA can also activate the NALP3 inflammasome leading to IL-1β secretion [46]. Canonical activation of the NALP3 inflammasome requires two sequential signals, priming, e.g., by LPS leading to the expression of pro-cytokines induced by NFkB, and activation by another stimulus [8,12,47]. Insoluble particles such as alum activate NALP3 via disruption of lysosomes and released cathepsin activity in the cytosol [7,12]. Interestingly, NETs have been shown to function as a priming signal, and subsequent stimulation with cholesterol crystals led to IL-1β production in macrophages [38]. In contrast, our study revealed that NET-priming led to unchanged or rather decreased cytokine production, whereas priming with LPS and activation with vaccine preparations induced higher levels of IL-1β or IL-6. Notably, priming of PBMC with LPS or NETs had an extremely donor-dependent effect on IL-1β production. The heterogeneity of innate immune responses by neutrophils and monocytes was striking, e.g., regarding cROS induction, expression of surface molecules or cytokines. Innate immune responses are known to vary individually. For instance, high and low cytokine responders to LPS or MPLA have been reported [48]. The variation of LPS-induced cytokines has been shown to be genetically inherited and is especially high for IL-1β with 86% heritability [49].

One limitation of this study is that the alum and MCT-containing AIT-vaccines used did not contain the same allergen extracts, which may additionally have contributed to the activation of monocytes. In principle, the in vivo effects of alum or alum-induced NETs may also involve the induction of chemokines and the recruitment of APCs, i.e., parameters we have not addressed in this study. However, alum apparently does not appear to induce considerable levels of chemokines in monocytes [23]. Moreover, additional factors such as complement and immune complexes may contribute in vivo to the activation of APCs.

## 5. Conclusions

Taken together, NETs induced by AIT-vaccines, alone or in acting together with alum, appear to promote the innate immune response to AIT-vaccines by affecting early events such as monocyte maturation into APC and antigen-uptake. Heterogenous response patterns to adjuvants and NETs in the population might at least, in part, explain the high incidence (~30%) of non-responders to AIT-vaccines.

## Data Availability

The data that support the findings of this study are available from the corresponding author upon reasonable request.

## Supplementary Materials

The following are available online at https://www.mdpi.com/article/10.3390/vaccines9040321/s1, Figure S1: Kinetics of DNA release induced by alum or MPLA, Figure S2: No DNA release induced by allergen extracts, Figure S3: Medium controls for monocyte staining after 24 h, Figure S4: Induction of IL-1β/IL-6 release in PBMC by LPS or NET priming or stimulation with complete vaccines, Video S1: Neutrophils in medium, Video S2: NET formation induced by ionomycin, Video S3: NET formation induced by alum, Video S4: Neutrophils in medium, Video S5: NET formation induced by ionomycin, Video S6: NET formation induced by alum, Video S7: NET formation induced by the alum-adjuvanted vaccine A-HDM, Video S8: NET formation induced by the alum-adjuvanted vaccine A-BP, Video S9: Neutrophils stimulated with MPLA, Video S10: Neutrophils stimulated with the MPLA/tyrosine microcrystals-adjuvanted vaccine MT-GP.
