# Supplementary material for: Adjuvants and Vaccines Used in Allergen-Specific Immunotherapy Induce Neutrophil Extracellular Traps
Source: Vaccines (Basel). 2021 Apr 1;9(4):321. doi: 10.3390/vaccines9040321 (PMC8066953; doi:10.3390/vaccines9040321)
Supplement: Supplementary file 1 [file vaccines-09-00321-s001.zip › Neuer Ordner/Supplementary material_0403.docx]

**Supplemental Videos - legends:**

**Video 1: Neutrophils in medium.** Neutrophils were primed with 25 ng/ml GM-CSF and incubated for 2 h in HBSS medium with Sytox green and monitored by fluorescence microscopy (bright field and green fluorescence channels merged; 100x objective; scale bar, 12 µm).

**Video 2: NET formation induced by ionomycin.** Neutrophils were primed with 25 ng/ml GM-CSF and incubated for 2 h with ionomycin (5 µM) with Sytox green in HBSS and monitored by fluorescence microscopy (bright field and green fluorescence channels merged; 100x objective; scale bar, 12 µm).

**Video 3: NET formation induced by alum.** Neutrophils were primed with 25 ng/ml GM-CSF and incubated for 2 h with alum (100 µg/ml) with Sytox green in HBSS and monitored by fluorescence microscopy (bright field and green fluorescence channels merged; 100x objective; scale bar, 12 µm).

**Video 4: Neutrophils in medium.** Neutrophils were primed with 25 ng/ml GM-CSF and incubated for 2 h in HBSS with Sytox green and monitored by fluorescence microscopy (bright field and green fluorescence channels merged; 20x objective; scale bar, 20 µm).

**Video 5: NET formation induced by ionomycin.** Neutrophils were primed with 25 ng/ml GM-CSF and incubated for 2 h with ionomycin (5 µM) with Sytox green in HBSS and monitored by fluorescence microscopy (bright field and green fluorescence channels merged; 20x objective; scale bar, 20 µm).

**Video 6: NET formation induced by alum.** Neutrophils were primed with 25 ng/ml GM-CSF and incubated for 2 h with alum (100 µg/ml) with Sytox green in HBSS and monitored by fluorescence microscopy (bright field and green fluorescence channels merged; 20x objective; scale bar, 20 µm).

**Video 7: NET formation induced by the alum-adjuvanted vaccine A-HDM.** Neutrophils were primed with 25 ng/ml GM-CSF and incubated for 2 h with the vaccine A-HDM (89 µg alum/ml) with Sytox green in HBSS and monitored by fluorescence microscopy (bright field and green fluorescence channels merged; 20x objective; scale bar, 20 µm).

**Video 8: NET formation induced by the alum-adjuvanted vaccine A-BP.** Neutrophils were primed with 25 ng/ml GM-CSF and incubated for 2h with the vaccine A-BP (100 µg alum/ml) with Sytox green in HBSS and monitored by fluorescence microscopy (bright field and green fluorescence channels merged; 20x objective; scale bar, 20 µm).

**Video 9: Neutrophils stimulated with MPLA.** Neutrophils were primed with 25 ng/ml GM-CSF and incubated for 2 h with MPLA (5 µg/ml) with Sytox green in HBSS and monitored by fluorescence microscopy (bright field and green fluorescence channels merged; 20x objective; scale bar, 20 µm).

**Video 10: Neutrophils stimulated with the MPLA/tyrosine microcrystals-adjuvanted vaccine MT-GP.** Neutrophils were primed with 25 ng/ml GM-CSF and incubated for 2 h with the vaccine MT-GP (2 µg MPLA/ml) with Sytox green in HBSS and monitored by fluorescence microscopy (bright field and green fluorescence channels merged; 20x objective; scale bar, 20 µm).

**Supplemental Figures**


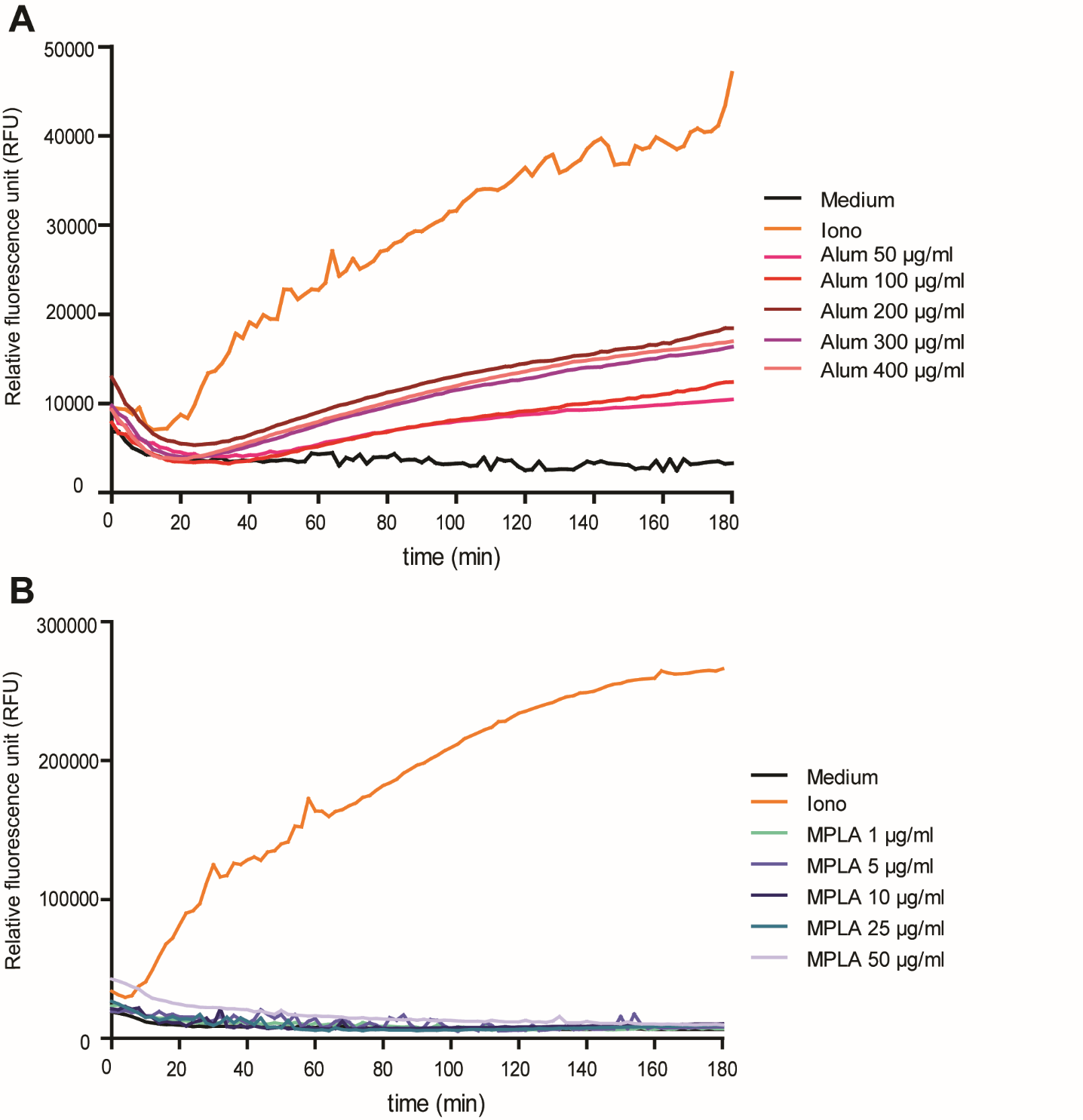


**Figure S1: Kinetics of DNA release induced by alum or MPLA.** Neutrophils were primed with 25 ng/ml GM-CSF and incubated for 3 h with medium, ionomycin (5 µM), alum (A) or MPLA (B) at the indicated concentrations. NET release was measured by fluorescence of Sytox orange bound to extracellular DNA in a plate reader assay.


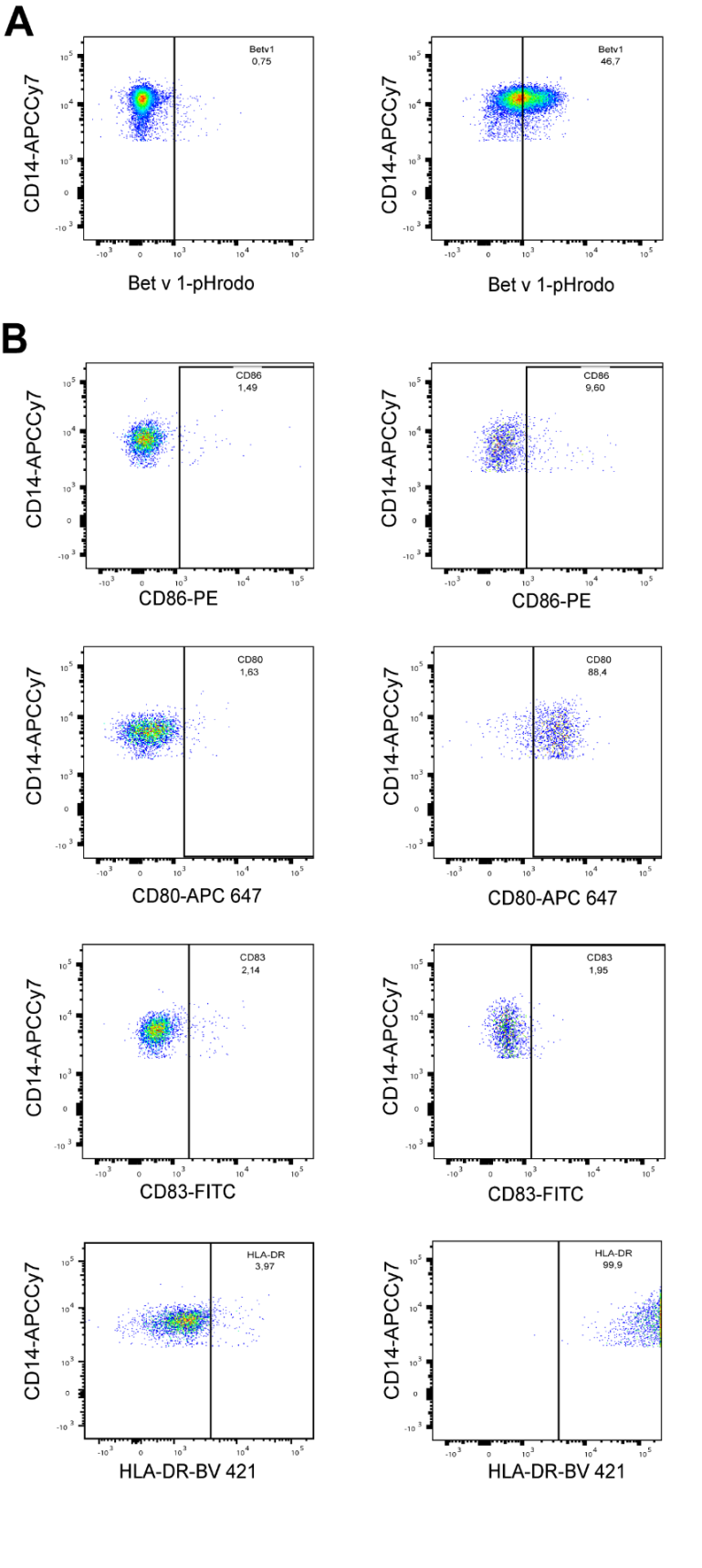


**Figure S2:** **Medium controls for monocyte staining after 24 h.** Medium control samples as reference for the expression of surface markers on monocytes within PBMC after 24 h incubation. (A) Fluorescence of CD14^+^ monocytes after 24 h of incubation of PBMC in medium (left panel) or in the presence of Bet v 1-pHrodo (1µg/ml) (right panel). (B) Isotype controls (left panels) and staining of CD14^+^ monocytes with specific antibodies (right panels) are shown.

**
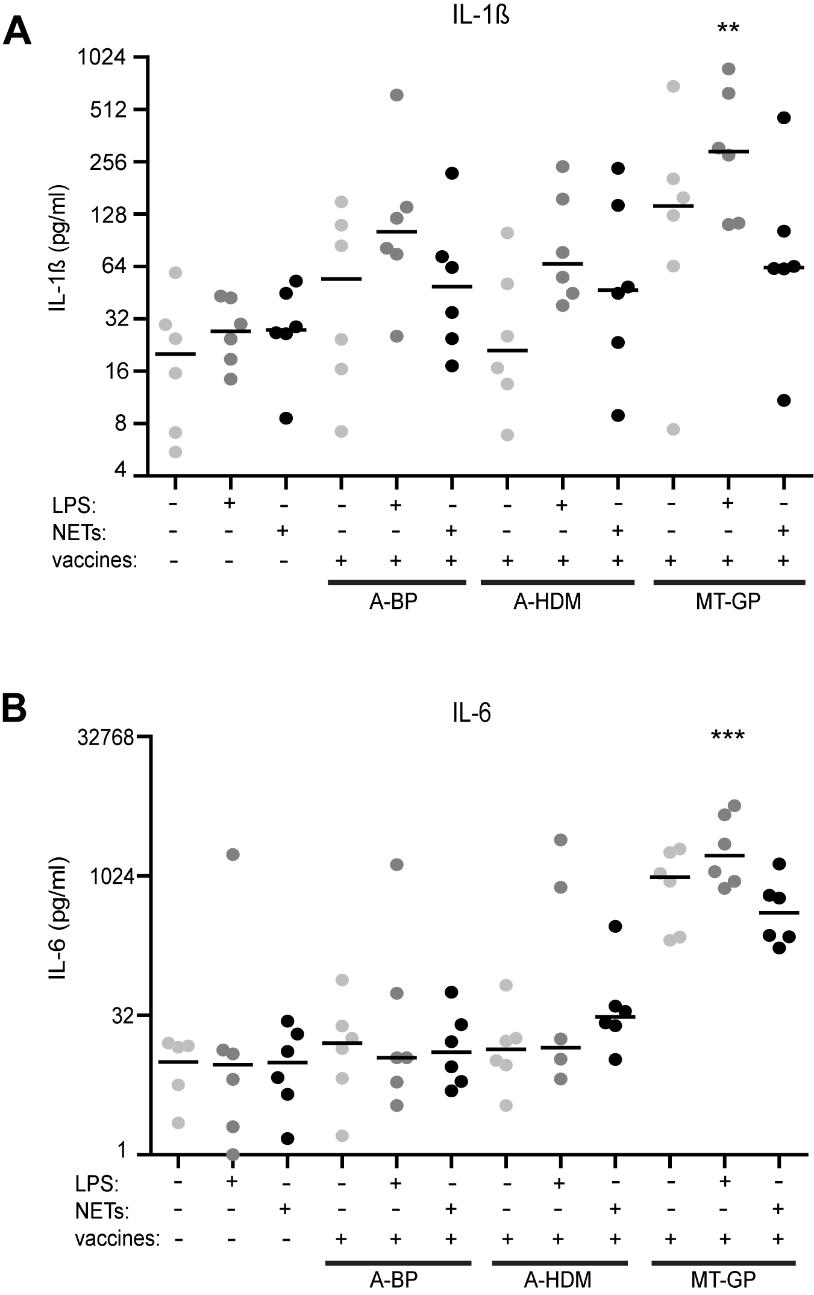
**

**Figure S3: Induction of IL-1β/IL-6 release in PBMC by LPS or NET priming or stimulation with complete vaccines.** PBMC were incubated for 3 h with either medium (neg. control), ultrapure LPS (25 pg/ml) or isolated NETs (100 ng/ml) and then stimulated for 6 h with either one of the alum-adjuvanted vaccines (A-HDM, A-BP) or the MPLA-adjuvanted one MT-GP. Cytokine levels of (A) IL-1β and (B) IL-6 in SN were measured by ELISA. Cumulative data of 3 independent experiments with 6 different donors are shown. One-way ANOVA followed by Dunnetts posttests (*p<0.05; **p<0.01; ***p<0.001).
